# Standardized Program of Resistance Training for Prostate Cancer Patients Receiving Androgen Deprivation Therapy (SPoRT-PCa-ADT): study protocol for a randomized controlled trial

**DOI:** 10.1186/s12885-026-15902-w

**Published:** 2026-04-22

**Authors:** Alejandro Soler-López, Carlos D. Gómez-Carmona, Daniel González-Devesa, Daniel López-Plaza, Laura Herrero-Vidal, Rubén Toledo-Pozuelo, Alberto González-Costea, Enrique Cao-Avellaneda

**Affiliations:** 1 OncoSport Never Surrender Foundation, Murcia, 30005 Spain; 2 Physical Activity and Sports Area, Department of General and Specific Didactics, Faculty of Education, University of Alicante, San Vicente del Raspeig, 03069 Spain; 3 Research Group in Training, Physical Activity and Sports Performance (ENFYRED), University of Zaragoza, Huesca, 50009 Spain; 4 Research Group in Optimization of Training and Sports Performance (GOERD), University of Extremadura, Caceres, 10005 Spain; 5 Research Group on Physical Activity, Education, and Health (GIAFES), Catholic University of Ávila, Ávila, 05005 Spain; 6 Urology Department of University Clinical Hospital “Virgen de la Arrixaca”, El Palmar, Murcia 30120 Spain

**Keywords:** Cancer-related fatigue; exercise oncology, Hypogonadism, Neuromuscular function, Supervised exercise intervention

## Abstract

**Background:**

Androgen Deprivation Therapy (ADT) for prostate cancer causes significant adverse effects including muscle wasting, bone density reduction, increased fatigue, and psychological distress. Despite growing evidence supporting exercise as an effective countermeasure, standardized evidence-based protocols for this population remain underdeveloped in clinical practice. Exercise is beneficial for managing cancer-related side effects, and general exercise guidelines for cancer survivors are available, but tailoring of exercise interventions may be more effective in those with treatment-specific complications.

**Methods/design:**

This randomized controlled trial with two parallel arms compares a 24-week supervised progressive resistance training program (SPoRT-PCa-ADT) with a control group receiving personalized home-based training following initial assessment and weekly telephone follow-up. The SPoRT-PCa-ADT Program utilizes velocity-based training methodology, progressing through three distinct phases (adaptation, development, maintenance) with intensity corresponding to 60% 1RM (mean propulsive velocity 0.92 m/s) and a 10% velocity loss threshold. The exercise protocol focuses primarily on squat movement patterns with real-time velocity feedback and incorporates daily readiness assessment to accommodate treatment-related fatigue fluctuations. Control group participants receive structured written instructions for home-based resistance training including warm-up and three effective sets of 12 repetitions of box squats, to be performed two to three times per week on non-consecutive days, with weekly telephone monitoring. The comprehensive assessment protocol includes progressive loading tests, vertical jump assessment, isometric strength measurement, and patient-reported outcomes measuring fatigue, quality of life, psychological distress, urinary function, and sleep quality. Assessments occur at baseline, midpoint (patient-reported outcomes only), and post-intervention, with additional biomarker collection.

**Discussion:**

To our knowledge, this will be the first study to implement a standardized velocity-based training protocol specifically developed for prostate cancer patients receiving ADT. The supervised program is delivered by exercise physiologists in a specialized sports center setting with consistent medical oversight, facilitating integration within cancer care pathways while ensuring exercise quality and safety. This detailed randomized controlled trial protocol provides clinicians with a standardized yet adaptable framework for exercise prescription in PCa-ADT patients, bridging the gap between evidence-based exercise principles and clinical implementation.

**Trial registration:**

ClinicalTrials.gov (NCT07064811, protocol version: 1.0, date registered:15 July 2025).

**Supplementary Information:**

The online version contains supplementary material available at 10.1186/s12885-026-15902-w.

## Background

Cancer remains a leading cause of mortality globally, with the World Health Organization reporting an estimated 10 million deaths annually [1]. This complex group of diseases comprises over 200 unique varieties, each presenting distinct challenges regarding management and treatment. The global cancer burden continues to rise, with an estimated 19.3 million new cases in 2020 and projections indicating this will increase to 28.4 million by 2040 [2]. Among the various cancer types, prostate cancer (PCa) represents a significant global health concern as the second most frequently diagnosed cancer in men worldwide. According to Global Cancer Observatory estimates, approximately 1.4 million new cases of PCa were diagnosed in 2020, with incidence rates projected to continue rising, particularly in developed countries [3].

Androgen Deprivation Therapy (ADT) has been established as a cornerstone treatment for advanced PCa, demonstrating considerable success in managing disease progression [4]. This treatment acts through multiple mechanisms to decrease testosterone levels, thereby inhibiting or slowing cancer growth. While effective as a cancer treatment, ADT induces a multifaceted set of adverse effects that substantially impact patients’ quality of life. As Crawford et al. documented [5], ADT-induced hypogonadism causes numerous physiological and psychological sequelae ranging from severe fatigue and muscle wasting to decreased bone density and psychological distress. These side effects typically persist throughout the entire treatment duration, which in many cases may extend for several years.

The physiological effects of ADT have been extensively investigated in the research literature. Smith et al. [6] demonstrated that individuals receiving ADT experience considerable decreases in muscle mass, with losses reaching 3–4% in the first year of treatment. This reduction in lean body mass is accompanied by decreases in strength and power output, variables directly linked to functional independence and quality of life [7]. Longitudinal research has shown that these changes can occur as early as three months following treatment initiation and continue to progress throughout therapy. Additionally, patients typically experience increased fat mass and decreased bone mineral density, making them susceptible to fractures and metabolic disease [8]. Research has demonstrated that bone loss during the first year of ADT can be up to 10-fold higher than bone loss from normal aging in healthy men.

The psychological impact of ADT is equally significant and interconnected with physical changes. A systematic review conducted by Qazi et al. [9] concluded that patients undergoing ADT develop considerable levels of fatigue, depression, sexual dysfunction, and anxiety. This combination of symptoms, also referred to as asthenia, represents a chronic condition of physical weakness and mental fatigue that severely limits patients’ capacity to conduct normal activities and maintain social relationships [10]. The psychological impact extends beyond the individual patient, affecting familial relationships, social connections, and overall quality of life. Research has established that psychological effects can persist even after treatment completion, highlighting the need for integrated support strategies.

Physical exercise has emerged as a promising strategy for reducing the side effects of ADT, supported by several systematic reviews and meta-analyses [11, 12]. Studies have demonstrated that structured exercise programs can preserve muscle mass, protect bone density, decrease fatigue, and enhance psychological well-being in this population. Nevertheless, despite the expanding evidence base, a crucial gap exists in standardized, evidence-based exercise guidelines specifically developed for this population. Although generic exercise recommendations are available for cancer patients [13], the distinct physiological and psychological sequelae experienced by men receiving ADT require more targeted interventions. Current literature demonstrates significant heterogeneity in exercise prescription parameters (e.g., supervised versus unsupervised, frequency, progression strategies), creating challenges for clinicians seeking to implement evidence-based practice [14].

The development of standardized exercise programs for specific clinical populations requires careful consideration of multiple factors, including baseline functional status, treatment-related limitations, and progression parameters [15]. Exercise programs for men receiving ADT must address both the physiological deconditioning and psychological impact of treatment while ensuring safety and feasibility. Previous protocols have demonstrated varied success, yet most lack the specificity and rigorous evaluation methods required for widespread clinical implementation [16]. Of particular importance are considerations regarding the timing of exercise initiation relative to ADT commencement, the balance between resistance and aerobic training components, and strategies for incorporating behavioral support interventions to enhance adherence [17].

Moreover, evaluation of exercise protocols must be supported by robust and standardized assessment methods. Current evaluation practices in this population demonstrate considerable heterogeneity, making cross-study comparisons challenging and hindering the translation of evidence-based treatments [18]. Physical evaluations must account for the unique limitations and risks associated with ADT, while psychological assessments must address the complex relationships between physical and mental health. A standardized assessment protocol would enable more consistent outcome measurement and facilitate better comparison of intervention effectiveness across diverse clinical settings.

Recent advances in exercise prescription methodology, particularly velocity-based training (VBT), offer promising opportunities for optimizing exercise interventions in clinical populations [19]. VBT allows for real-time monitoring and adjustment of training loads based on movement velocity, providing a more individualized and responsive approach to exercise prescription than traditional percentage-based methods. This methodology may be particularly relevant for ADT patients who experience significant day-to-day variations in energy levels and physical capacity due to treatment-related side effects.

Therefore, the purpose of this manuscript is to describe a comprehensive exercise protocol and evaluation system specifically designed for prostate cancer patients receiving androgen deprivation therapy (SPoRT-PCa-ADT). The protocol was developed based on the existing evidence regarding exercise prescription for cancer populations and specifically addresses the limitations associated with patients undergoing androgen deprivation therapy [20]. Furthermore, the evaluation system incorporates validated instruments and standardized procedures, providing a systematic methodology for assessing both physical and psychosocial outcomes in this population. The specific objectives are to: (a) present a comprehensive, evidence-based exercise protocol addressing both physical and psychological consequences of ADT; (b) describe a detailed evaluation methodology for this population; (c) provide explicit implementation guidelines including progression criteria and safety considerations; and (d) establish standardized outcome measures for assessing protocol effectiveness, ultimately bridging the gap between research evidence and clinical practice.

## Methods

### Patient and public involvement

The design of the SPoRT-PCa-ADT trial was informed by the involvement of the Never Surrender Foundation (OncoSport Never Surrender Foundation, Murcia, Spain), a patient advocacy organization dedicated to improving the health and quality of life of cancer patients through exercise-based programs. Representatives of the Foundation participated in the conceptualization of the intervention model, including the selection of the exercise setting, the structure of the supervised sessions, and the design of the home-based control program, ensuring that the protocol reflects the practical needs and preferences of prostate cancer patients undergoing ADT. Additionally, patient perspectives informed the choice of patient-reported outcome measures included in the assessment protocol, prioritizing instruments addressing the symptoms most frequently reported as burdensome by this population, namely fatigue, urinary function, and psychological distress. Patients were not directly involved in the statistical analysis plan or the preparation of this manuscript. Results will be disseminated to participants and the broader patient community through the Never Surrender Foundation upon study completion.

### Design

**Fig. 1:**
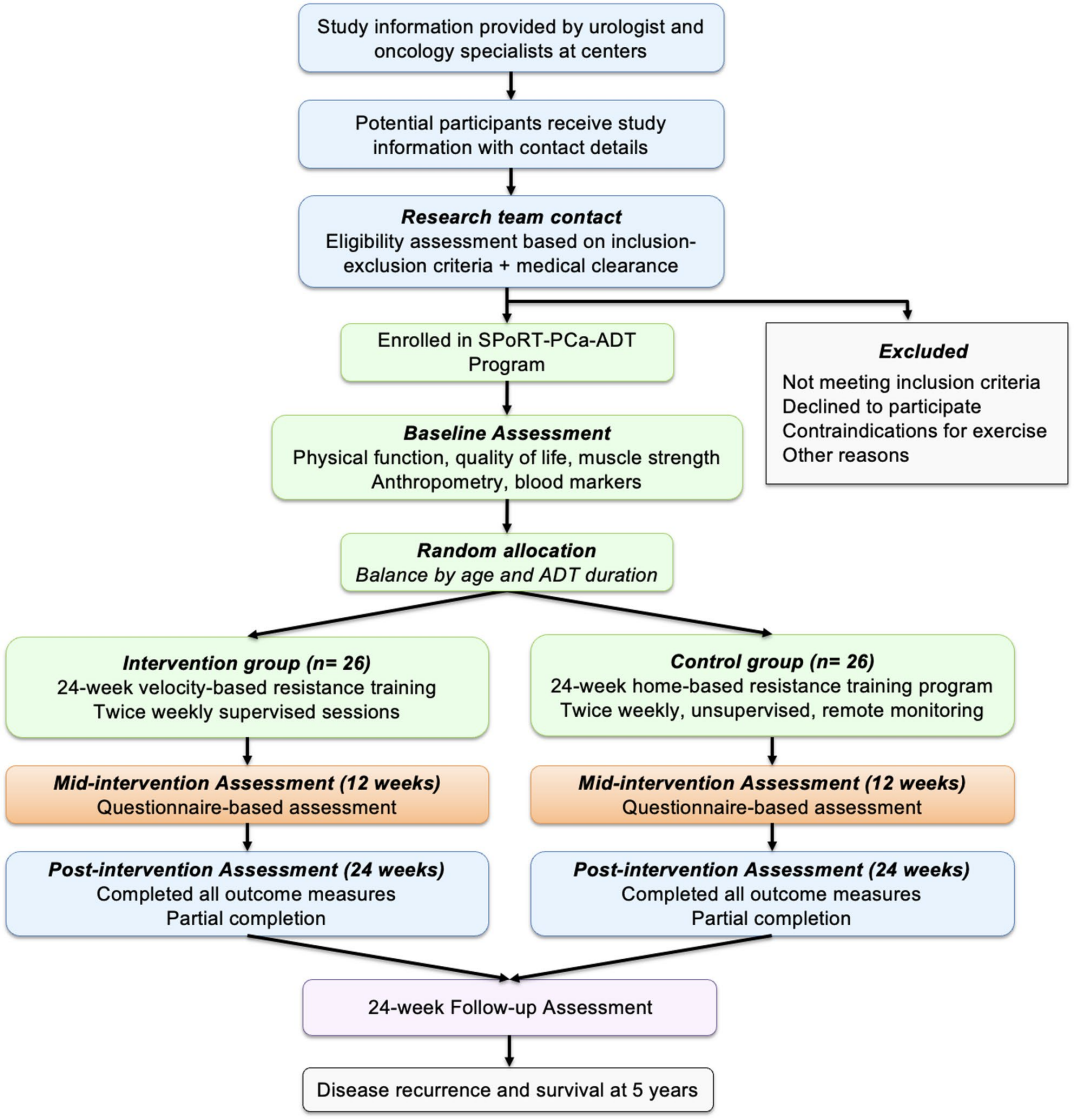
Patient Flowchart of the SPORT-PCa-ADT Program

This randomized controlled trial (RCT) with two parallel study arms evaluates the effectiveness of a 24-week exercise intervention in prostate cancer (PCa) patients undergoing androgen deprivation therapy (ADT). Ethical approval was obtained from the Clinical Research Ethics Committee of the University Clinical Hospital “Virgen de la Arrixaca” (Murcia, Spain) (ID: 2024-5-7-HCUVA, approval date: 28 May 2024). Participants are randomly assigned to one of two groups: (1) a supervised progressive resistance training program (SPoRT-PCa-ADT), or (2) a control group receiving a home-based exercise protocol following an initial assessment, accompanied by weekly telephone follow-up. Assessments are conducted at baseline, 12 and 24 weeks to examine changes in physical, functional, and psychosocial outcomes. Additionally, patient-reported outcomes are collected at the 12-week midpoint (Fig. 1). This study design enables the evaluation of both immediate and sustained intervention effects and facilitates a direct comparison between supervised and home-based exercise approaches, in line with methodological recommendations proposed by Galvão et al. [20].

The theoretical framework integrates progressive resistance training principles with specific considerations for ADT-induced physiological changes, including muscle loss, increased fat mass, and decreased bone mineral density. The program emphasizes velocity-based training methods, functional movement patterns, systematic progression accounting for treatment-related symptoms, and evidence-based assessment tools appropriate for this population, aligning with community-based exercise programs that have demonstrated effectiveness for cancer patients as described by Cormie et al. [21]. The program is implemented by the Never Surrender Foundation in collaboration with the Urology Department of the University Clinical Hospital “Virgen de la Arrixaca” in the Region of Murcia, Spain.

Any important modifications to the trial protocol (e.g., changes to eligibility criteria, outcomes, or statistical analyses) will require prior approval from the Clinical Research Ethics Committee of the University Clinical Hospital ‘Virgen de la Arrixaca’. Approved amendments will be updated on ClinicalTrials.gov (NCT07064811) and communicated to the research team and, where applicable, to enrolled participants. Participants whose continued trial participation is affected by an amendment will be re-consented using an updated consent form.

### Participants

#### Eligibility criteria

Male individuals of legal age diagnosed with locally advanced or metastatic prostate cancer and undergoing androgen deprivation therapy (ADT) are eligible for inclusion in this randomized controlled trial. The primary objective was to assess the impact of a structured physical exercise intervention on physical function, cancer-related fatigue, and quality of life in this patient population. Participants are randomly allocated to one of two groups: those in the intervention arm received supervised exercise in accordance with the SPoRT-PCa-ADT Program, while those in the control arm are provided with a home-based exercise regimen following an initial evaluation, supported by weekly telephone follow-ups.

The inclusion and exclusion criteria are presented in the Table 1, following the recommendations of previous randomized controlled trials to target an clinically homogeneous population, maximizing potential benefits and be consistent with exercise guidelines for cancer patients [8, 22].

**Table 1:** Inclusion and exclusion criteria

Inclusion criteria	Male participants aged 18–80 years Diagnosis of locally advanced or metastatic prostate cancer Currently receiving androgen deprivation therapy (ADT) Treatment at Urology and oncology departments within the University Clinical Hospital “Virgen de la Arrixaca” healthcare network Legal capacity and signed informed consent Ability to attend supervised sessions or perform home-based exercises safely Physician clearance for exercise participation
*Exclusion criteria*	Unwillingness to participate in the study Inability to complete adequate follow-up Uncontrolled or symptomatic metastatic bone disease Physical disabilities that would prevent participants from safely completing the protocol Severe cardiovascular disease or contraindications to exercise determined by assessment Participation in structured resistance training within the previous 6 months Cognitive impairment limiting ability to follow exercise instructions Uncontrolled hypertension (> 160/100 mmHg) Active treatment for other primary cancers

#### Sample size

Sample size calculations are performed using G*Power 3.1.9.7 software [23]. For the primary analysis examining the time × treatment interaction effect, an F-test for ANOVA: repeated measures, within-between interaction is selected. Parameters are set as follows: effect size f = 0.25 (converted from Cohen’s d = 0.5), α error probability = 0.05, power (1-β error probability) = 0.95, number of groups = 2, number of measurements = 3 (baseline, midpoint, post-intervention), correlation among repeated measures = 0.5 (estimated from previous resistance training studies in cancer populations), and nonsphericity correction ε = 1. The moderate effect size (Cohen’s d = 0.5) is selected based on previous research demonstrating moderate to large effects of resistance training interventions on muscle strength and functional outcomes in ADT patients [24, 25]. This analysis determines that 44 participants (22 per group) provide adequate statistical power to detect the hypothesized interaction effect for the primary outcome measures. Accounting for an anticipated 15% dropout rate, based on previous reports from supervised exercise interventions in cancer survivors, 52 participants are targeted for recruitment (26 per group randomized to either the supervised velocity-based training program or the home-based control intervention).

#### Recruitment and screening

Potential participants are identified and enrolled through a systematic referral process from urology and oncology services at hospitals throughout the Region of Murcia. Multiple approaches are adopted to promote the program and facilitate recruitment, including training Urologists specialized in prostate cancer and radiation oncologists to provide direct patient referrals; distributing program brochures in hospitals, urology and oncology centers, and community organizations; email and postal communications to individuals who had previously contacted the Never Surrender foundation expressing interest in exercise programs; local media advertising; and dissemination through social media.

Upon initial contact, potential participants receive an information package containing detailed program information, screening documentation, and contact details for their corresponding program center. The screening process includes physician approval to ensure participant safety. Each participant´s urologist, oncologist or general practitioner completes a screening form confirming the absence of contraindications to exercise. This approach to medical clearance follows best practice recommendations for exercise program implementation in clinical populations [26].

#### Randomization

Following eligibility confirmation and informed consent, all participants complete baseline assessments prior to randomization. Participants are randomly allocated to groups using a computer-generated randomization sequence with variable block sizes (4, 6, and 8) to ensure allocation concealment and prevent prediction of future allocations [27]. Randomization is stratified by age group (< 65 vs. ≥65 years) and ADT duration (< 12 vs. ≥12 months) to ensure balanced distribution of key prognostic factors that may influence treatment response. Age stratification accounts for potential differences in exercise adaptation between younger and older adults [28], while ADT duration stratification addresses the progressive nature of treatment-related side effects, as longer ADT exposure is associated with greater muscle mass loss and functional decline [29]. An independent researcher with no involvement in participant recruitment or assessment performs the randomization procedure using a secure online randomization system.

#### Blinding

Due to the nature of the exercise intervention, blinding of participants and exercise physiologists is not feasible, which is consistent with the inherent limitations of behavioral intervention trials [30]. However, outcome assessors for objective physical measures remain blinded to group allocation where possible to minimize detection bias. All patient-reported outcome measures are self-administered electronically to eliminate potential assessor influence. Data files are anonymized using coded identifiers by an independent researcher prior to processing by study personnel, and data analysis will be conducted by statisticians blinded to group allocation [31]. To minimize performance bias, participants are informed that there is equal possibility of assignment to either treatment group, and that the relative effectiveness of two different exercise interventions is under investigation, without suggesting superiority of either approach.

### Exercise protocol of intervention group

The SPORT-PCa-ADT Program is designed following international guidelines on evidence-based exercise prescription for prostate cancer (PCa) patients undergoing ADT [13, 18]. The program is specifically tailored to counteract the well-documented adverse effects of ADT, including muscle loss, increased fat mass, decreased bone mineral density, and cancer-related fatigue. Particular attention was given to balancing physiological effectiveness with practical implementation considerations.

**Fig. 2:**
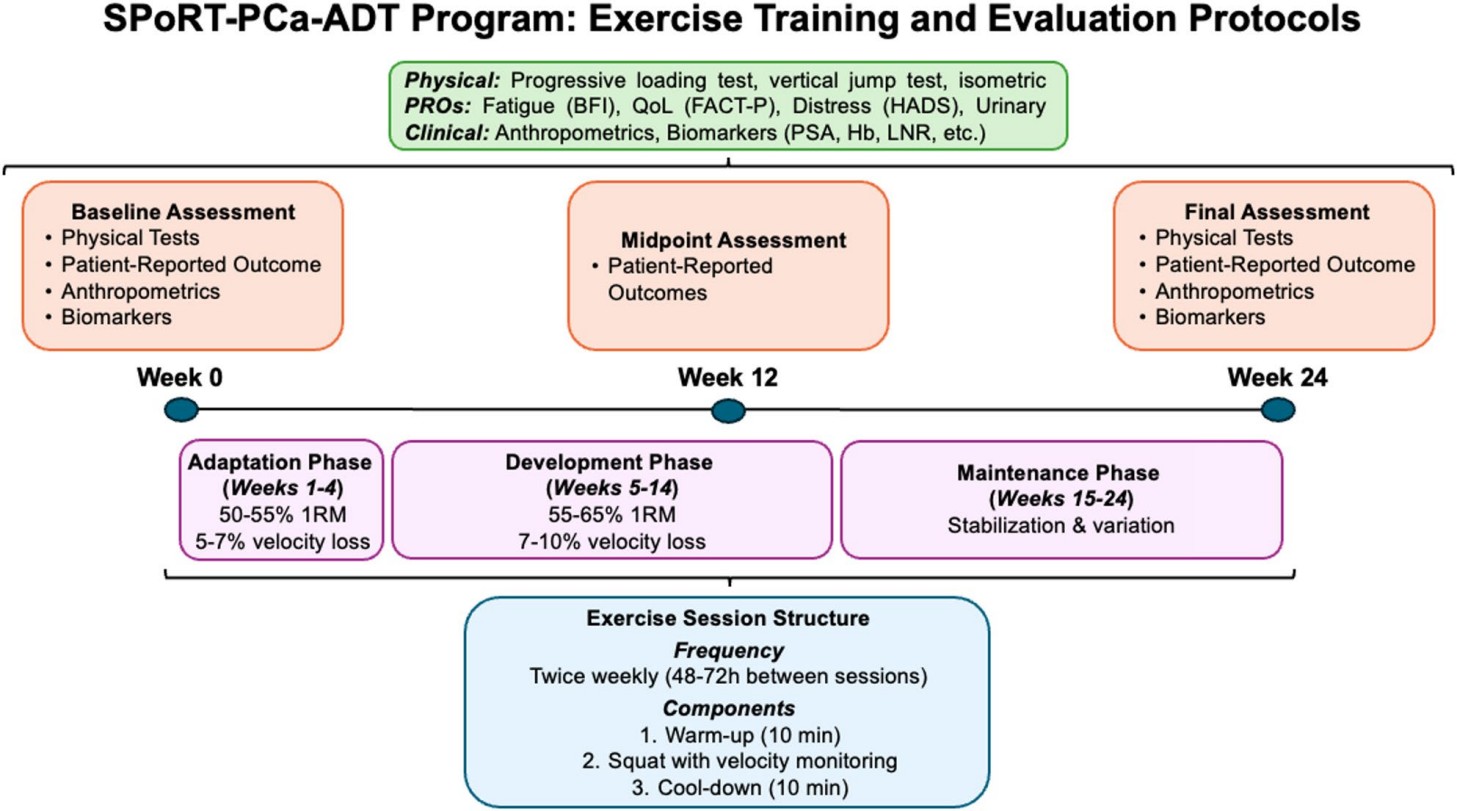
Exercise Training Sport Protocol Intervention and Evaluation Protocols in SPoRT-PCa-ADT

Subjects participate in the supervised exercise program twice weekly (with a 48–72-hour interval between sessions) for 24 weeks, totaling 48 supervised sessions. The progressive resistance training program uses velocity-based training principles [32–34], a novel approach in exercise oncology that allows for precise load management and individualization based on daily performance capacity [35]. The sessions are conducted in a specialized sports center under direct supervision of exercise physiologists and sports scientists specifically trained in the SPORT Protocol, at the same time of day (± 1 h) for each participant, and under controlled environmental conditions (20 °C and 60% humidity). The study protocol of exercise training and evaluation is shown in Fig. 2.

#### Exercise prescription

The primary resistance exercise focuses on the squat movement pattern due to its functional relevance and capacity to engage large muscle groups significantly affected by ADT [36]. The relative intensity of the exercise is calculated using velocity-based training, with each participant training at a load corresponding to 60% of 1-repetition maximum (1RM), equating to a mean propulsive velocity of 0.92 m/s [37]. A velocity loss threshold of 10% per exercise set is established—once this threshold is exceeded, the set is finished to prevent excessive fatigue and maintain training quality [32].

Each training session includes three effective sets per participant, resulting in a total of 144 effective sets per subject throughout the program (3 sets × 48 sessions). This approach ensures standardization of training volume while accommodating individual variations in strength and fatigue. Specific components of each session include:

1. A standardized warm-up consisting of 5 min of treadmill running at 4 km/h, 5 min of lower body joint mobilization exercises, and two preparatory sets of six squats with 6 kg loads (with 3-minute rests between sets).
2. The main resistance training component focuses on squat exercises using a Smith machine (without a counterbalance mechanism). Participants perform the squat starting from an upright position with knees and hips fully extended, feet parallel and shoulder-width apart, and the bar resting on the upper back at the acromion level. The descent phase is performed at a controlled mean velocity (~ 0.50–0.65 m/s) until the top of the thighs are below the horizontal plane, with posterior thighs and shins in contact (~ 35–40° knee flexion). The concentric phase is executed at maximum intended velocity.
3. Recovery periods between sets are strictly monitored, ranging from 3 min (for lighter loads) to 5 min (for heavier loads), to ensure adequate recovery of the neuromuscular system.
4. A 10-minute cool-down period includes light aerobic activity and dynamic stretching of the major muscle groups utilized during the session.

The dynamic measurement system (T-Force, Ergotech Consulting, Spain) provides real-time auditory and visual velocity feedback during each repetition, allowing participants to maintain the target velocity and enabling precise implementation of the velocity loss threshold. This system automatically calculated relevant kinematic parameters for each repetition and stored data for analysis [38].

#### Exercise progression and modification

While the program focuses primarily on squat exercises, supplementary exercises are incorporated based on individual participant needs and progression as determined by the supervising exercise physiologist. The program follows a periodized progression model with three distinct phases.

1. *Adaptation Phase (Weeks 1–4)* emphasized proper technique, movement patterns, and development of a basic strength foundation. During this initial phase, participants worked at lower intensities (50–55% of 1RM, corresponding to a mean propulsive velocity of ~ 1.0 m/s) with higher repetitions and lower velocity loss thresholds (5–7%). This conservative approach allows participants to develop technical proficiency while minimizing fatigue and soreness, particularly important for deconditioned individuals beginning an exercise program during cancer treatment.
2. *Development Phase (Weeks 5–14)* introduced a progressive increase in training load (55–65% of 1RM, ~ 0.92 –0.85 m/s) and velocity loss thresholds (7–10%) to drive physiological adaptations. This middle phase constitutes the core of the intervention, where the greatest improvements in muscular strength, physical function, and body composition are expected to occur. The gradual progression in intensity during this phase is carefully monitored to ensure that adaptations occurred without excessive fatigue or risk of injury.
3. *Maintenance Phase (Weeks 15–24)* focused on stabilization of training load with strategic variation in exercise selection and training modalities to prevent plateaus while maintaining adaptations. This final phase is designed to consolidate gains made during the development phase and establish sustainable long-term exercise behaviors. Small variations in exercise prescription are introduced to maintain participant engagement and prevent psychological fatigue from program monotony.

Throughout all phases, individualized modifications are made based on multiple factors. Individual response to each training session is carefully monitored through performance metrics and subjective feedback. The presence and severity of treatment-related side effects are assessed at each session, allowing for day-to-day adjustments in exercise prescription. Any pre-existing musculoskeletal conditions are accommodated through exercise modifications while maintaining program integrity. The rate of progression in strength and movement velocity is tracked longitudinally to ensure appropriate progression for each participant.

To accommodate individual variations in fatigue (particularly relevant in cancer patients), a daily readiness assessment is conducted before each session. This includes a subjective fatigue rating, measurement of resting heart rate, and performance of submaximal squat repetitions to assess movement velocity. If significant deviations from baseline are observed, the training load was adjusted accordingly to prevent overtraining while ensuring sufficient stimulus for adaptation. This responsive approach to exercise prescription allows the program to accommodate the fluctuating energy levels and symptoms commonly experienced by prostate cancer patients undergoing ADT. Additionally, we have monthly telematic meetings and direct consultations with the responsible medical team to address any doubts regarding changes in clinical status.

### Exercise protocol of control group

Participants assigned to the control group received structured written instructions for a personalized home-based resistance training program. The protocol is designed to prioritize safety, progressive adaptation, and individualization based on each participant’s functional capacity and available resources at home. The program is intended to be performed two to three times per week on non-consecutive days, focusing exclusively on lower-body training through a single primary movement. Each session consists of three effective sets of 12 repetitions, which serves as the standard training volume for the main exercise.

Each session begins with a standardized warm-up targeting the lower limbs, consisting of two sets of 15 repetitions each of seated knee extensions, seated ankle dorsiflexion and plantarflexion, and bodyweight glute bridges. Following the warm-up, participants perform box squats as the sole main exercise. This movement is chosen for its safety, simplicity, and suitability for progressive overload in a home setting. Participants are instructed to perform the concentric phase of each repetition with maximal voluntary velocity, a core element of the intervention aimed at optimizing neuromuscular engagement while limiting cumulative fatigue.

To ensure a low perception of effort and avoid excessive neuromuscular strain, the program prescribes a low-to-moderate training load, with each set performed with at least twice the number of repetitions in reserve relative to those completed. This approach is designed to maintain a subjective exertion level below 12 on the Borg 6–20 scale and to minimize velocity loss within each set. Participants are encouraged to use bodyweight or accessible household items as external resistance, depending on their capabilities and context.

Weekly telephone follow-ups are conducted by trained research staff to reinforce adherence, provide feedback, and resolve any concerns. Additionally, participants are provided with a structured training log to self-monitor their sessions and support continued engagement with the program.

### Concomitant care

All participants, regardless of group allocation, are permitted and encouraged to continue all ongoing oncological treatments as prescribed by their responsible urologist or oncologist, including androgen deprivation therapy, radiotherapy, or any other systemic or supportive treatments. No modifications to participants’ oncological treatment regimens will be made because of trial participation. Participants are asked to refrain from engaging in any additional structured resistance training program outside of the trial protocol during the 24-week intervention period, in order to avoid confounding the specific effects of the allocated exercise intervention. Unstructured physical activity of low-to-moderate intensity, such as walking or activities of daily living, is permitted and will be recorded through self-report at each assessment timepoint. Any change in oncological treatment or initiation of new pharmacological agents during the trial period will be documented and considered in the sensitivity analyses.

### Assessment protocol

All participants undergo identical assessment procedures irrespective of group allocation. Baseline assessments are conducted within two weeks prior to randomization, and post-intervention assessments are scheduled within two weeks following completion of the 24-week intervention period. Midpoint assessments of patient-reported outcomes are conducted at week 12 and are administered either at the training center or during specialized urological consultations as part of routine oncological follow-up. To reduce the influence of diurnal variation, all physical performance assessments are scheduled at the same time of day for each participant (± 1 h). Participants are instructed to refrain from strenuous physical activity for 48 h prior to testing, maintain adequate hydration, and abstain from caffeine and alcohol for 24 h before each assessment (Fig. 2).

Physical assessments are conducted by qualified sports scientists with training in exercise oncology. Due to logistical constraints, assessors are not blinded to group allocation. However, patient-reported outcomes are collected using standardized, self-administered questionnaires to mitigate potential assessor bias. Venous blood samples are drawn by certified phlebotomists and analyzed by laboratory technicians blinded to group assignment. To ensure procedural consistency, comprehensive standard operating procedures (SOPs) are developed prior to study initiation, and all research personnel complete structured training. Ongoing quality control procedures are implemented throughout the study to uphold methodological rigor.

#### Physical assessments

##### Progressive loading test

A progressive loading test is conducted using the squat (SQ) exercise to evaluate strength and velocity parameters. Participants perform the SQ starting from an upright position with knees and hips fully extended, feet parallel and shoulder-width apart, and the bar resting on the upper back at the acromion level. Each participant descends continuously until the top of the thighs were below the horizontal plane, with the posterior thighs and shins in contact (~ 35–40° knee flexion), then immediately reverses the movement to return to the upright position.

Unlike the eccentric phase, which is performed at a controlled mean velocity (~ 0.50–0.65 m/s), participants are required to execute the concentric phase at maximum intended velocity. The initial load is set at 6 kg and progressively increased in 5 kg increments until the mean propulsive velocity (MPV) reached 70% of 1RM. Strong verbal encouragement is provided to motivate participants to exert maximum effort. Recovery times between sets vary from 3 min (light loads) to 5 min (heavy loads). Only the best repetition at each load, based on the fastest MPV criteria, is considered for subsequent analysis.

All velocity measurements reported in this study correspond to the mean velocity of the propulsive phase of each repetition [37]. The propulsive phase is defined as the fraction of the concentric phase during which the bar’s acceleration exceeded gravitational acceleration [39]. A Smith machine without a counterbalance mechanism is used for testing and training. A dynamic measurement system (T-Force, Ergotech, Spain) automatically calculates relevant kinematic parameters for each repetition, provides real-time auditory and visual velocity feedback, and stores data for analysis.

##### Vertical jump test

In the countermovement jump (CMJ) test, participants perform five maximum CMJs with 20-second rests between jumps. Before CMJ evaluation, participants warm up by performing two sets of 10 squats without external load, 5 submaximal CMJs, and 3 maximum CMJs. CMJ height was calculated from flight time values determined using a contact platform (Chronojump Boscosystem^®^, Barcelona, Spain). After discarding the highest and lowest CMJ heights, the resulting average is retained for further analysis. This test serves as a measure of lower body power and neuromuscular function, which are commonly impaired in patients undergoing ADT.

##### Maximum isometric contraction test

Maximum isometric force (MIF) and maximum rate of force development (RFDmax) are measured during a maximum voluntary isometric contraction (MVIC) test in the squat (SQ) exercise with participants standing and knees flexed at 90° (180° = full extension). This test is performed on a quadriceps extension machine. Participants are instructed to push against the force platform as quickly and forcefully as possible following the “ready, set, go!” signal during two 5-second trials separated by 1-minute rests. External forces are recorded at a sampling rate of 1000 Hz and processed using specific software (Chronojump Boscosystem^®^, Barcelona, Spain). RFDmax is calculated as the maximum slope in the time-force curve over 20 ms intervals. Additionally, the average tangential slope of the time-force curve is obtained at different time intervals (50, 100, and 150 ms from the onset of force production, RFD0-50, RFD0-100, and RFD0-150, respectively) was calculated. The average value of each variable from the two attempts is recorded.

#### Patient-reported outcomes

- *Fatigue assessment*: Cancer-related fatigue, one of the most common and debilitating side effects of cancer treatment and ADT, is assessed using the Brief Fatigue Inventory Short form (BFI-SF). This validated 9-item questionnaire measures the severity of fatigue and its interference with daily activities. Scores are categorized as mild (1–3), moderate (4–6), or severe (7–10) fatigue. The BFI-SF sf has demonstrated excellent reliability and validity in cancer populations and is sensitive to changes following exercise interventions [40].
- *Quality of Life Assessment*: Quality of life is evaluated using the Functional Assessment of Cancer Therapy-Prostate (FACT-P) questionnaire. This 39-item instrument comprises the FACT-General (FACT-G) plus a prostate cancer subscale (PCS) that assesses concerns specific to prostate cancer and its treatment. The FACT-P evaluates physical wellbeing, social/family wellbeing, emotional wellbeing, functional wellbeing, and prostate cancer-specific concerns. Higher scores indicate better quality of life. This instrument has been extensively validated in prostate cancer populations and demonstrates good sensitivity to change following interventions [41].
- *Psychological Distress*: Psychological distress is assessed using the Hospital Anxiety and Depression Scale (HADS), a 14-item questionnaire designed to identify anxiety and depression in patients with physical health problems. The scale produces separate scores for anxiety and depression. The HADS has been validated in cancer populations and is widely used in psycho-oncology research [42].
- *Urinary Function*: Urinary function, which can be affected by both prostate cancer and its treatments, is assessed using the International Prostate Symptom Score (IPSS). This 8-item questionnaire evaluates the severity of lower urinary tract symptoms associated with benign prostatic hyperplasia and other conditions affecting urinary function in men. The total symptom score ranges from 0 to 35, with higher scores indicating more severe symptoms. Additionally, includes an ancillary query regarding the patient’s quality of life related to urinary symptoms, thereby providing a comprehensive evaluation that supports both diagnostic and therapeutic decision-making in clinical practice [43].
- *Sleep Quality*: Sleep disturbances are common in cancer patients and can significantly impact quality of life. Sleep quality is assessed using the Pittsburgh Sleep Quality Index (PSQI), a 19-item self-report questionnaire that evaluates sleep quality and disturbances over a one-month period [44]. The PSQI generates seven component scores and a global score from 0 to 21, with higher scores indicating worse sleep quality.

#### Additional clinical measures

- *Anthropometrics*: Height is measured to the nearest 0.1 cm using a wall-mounted stadiometer. Weight and body composition are measured through an 8-electrode segmental monitor (TANITA, Tokyo, Japan). In addition, body mass index (BMI) is calculated as weight (kg) divided by height squared (m²). All measurements are taken in duplicate, and the average are used for analysis.
- *Biomarkers*: Blood samples are collected after an overnight fast at baseline and post-intervention to assess biomarkers relevant to prostate cancer and exercise response. Prostate-specific antigen (PSA), hemoglobin (Hb), lymphocyte-to-neutrophil ratio (LNR), and alkaline phosphatase levels (LDH) are analyzed. Additionally, to evaluate exercise-induced changes in oxidative stress and inflammatory markers, high-sensitivity C-reactive protein (hsCRP) and interleukin-6 (IL-6) are assessed. All samples are processed and analyzed according to standardized procedures.

### Program monitoring and safety

The incidence and severity of adverse events during exercise sessions are systematically monitored and reported by supervising exercise physiologists using standardized documentation. Events are classified by type and severity, with participants also self-reporting any adverse events between sessions. A Data Safety Monitoring Board reviews all events to ensure participant safety, with serious adverse events are reported to the institutional Ethics Committee immediately upon identification and formally documented within 24 h.

Exercise adherence (percentage of scheduled sessions attended) and protocol compliance (actual loads, repetitions, and velocity parameters) are tracked to calculate total training volume and intensity received by each participant. Program adherence of ≥ 85% is required for per-protocol analysis, with strategies to maximize adherence including follow-up calls, flexible scheduling, and transportation assistance when feasible. Participants can withdraw at any time without prejudice to their ongoing care, and investigators can withdraw participants if they meet exclusion criteria or if continued participation is deemed potentially harmful.

No formal interim efficacy analyses are planned for this trial. Given the nature of the intervention and the study population, no independent Data Safety Monitoring Board (DSMB) has been constituted. Safety monitoring will be conducted by the principal investigator and the supervising exercise physiologists throughout the 24-week intervention period. Early termination of the trial may be decided by the principal investigator in consultation with the Clinical Research Ethics Committee of the University Clinical Hospital ‘Virgen de la Arrixaca’ if: (i) there is clear evidence of harm attributable to either intervention; (ii) enrolment falls below 50% of the target sample after 12 months of active recruitment; or (iii) continued participation is deemed clinically unjustifiable. All decisions regarding early stopping and their rationale will be formally documented.

Upon completion of the 24-week intervention, all participants — including those in the control group — will receive an individualised exercise prescription developed by the supervising exercise physiologists, based on their post-intervention assessment results. This provision ensures equitable access to evidence-based exercise support regardless of group allocation and is consistent with the ethical principle of beneficence in clinical trial design. In the event of a participant suffering harm directly attributable to trial participation, standard institutional indemnity provisions of the University Clinical Hospital ‘Virgen de la Arrixaca’ will apply. No additional financial compensation beyond standard clinical care is foreseen.

### Statistical analyses

Statistical analyses will be performed using JAMOVI (version 2.5, The JAMOVI Project, Sydney, Australia). Descriptive statistics will be calculated for all variables, with data presented as means and standard deviations for continuous variables and frequencies and percentages for categorical variables. Graphical representations will be generated using GraphPad Prism (version 10, GraphPad Software Inc., San Diego, CA, USA).

The primary analysis will include all participants who completed both baseline and post-intervention assessments in their respective groups. Additional sensitivity analyses will be conducted using intention-to-treat principles, with missing data handled using multiple imputation by chained equations (MICE) with predictive mean matching for continuous variables. For all outcome measures, analysis of covariance (ANCOVA) will be used to compare post-intervention values between groups while adjusting for baseline values and stratification variables (age group and ADT duration).

Bonferroni post-hoc corrections will be applied for multiple comparisons across outcome measures. Effect sizes will be calculated using Cohen’s d for between-group differences and partial eta-squared for ANCOVA models, with values of 0.2, 0.5, and 0.8 considered small, medium, and large effects for Cohen’s d, respectively [45]. For all analyses, *p* < 0.05 will be considered statistically significant. Per-protocol analyses will be conducted as sensitivity analyses, including only participants with ≥ 85% adherence. Exploratory subgroup analyses will examine potential effect modification by baseline characteristics including age, ADT duration, and baseline functional status. Correlation analyses will examine relationships between changes in objective physical performance measures and patient-reported outcomes within each group.

### Dissemination policy

The results of the SPoRT-Pca-ADT trial will be disseminated regardless of the direction or magnitude of findings. Primary outcomes will be submitted for publication in peer-reviewed journals in the fields of exercise oncology and urology, with secondary outcomes reported in subsequent publications. Findings will also be presented at relevant national and international scientific conferences. A summary of results will be communicated to participants upon study completion and shared with the prostate cancer patient community through the Never Surrender Foundation and the referring clinical teams at the Urology Department of the University Clinical Hospital ‘Virgen de la Arrixaca’. Summary results will be posted on ClinicalTrials.gov (NCT07064811) within 12 months of trial completion.

## Discussion

The SPoRT-PCa-ADT Program, now assessed against a concurrently evaluated SPoRT-Control Intervention group, presents a standardized exercise intervention specifically designed to address the adverse effects experienced by prostate cancer patients undergoing androgen deprivation therapy with specific assessments to guide the process. The protocol and its implementation reflect an evolving approach to exercise prescription in cancer care that warrants comparison with existing methodologies in the literature.

The velocity-based training (VBT) methodology incorporated in the SPoRT-PCa-ADT program represents a fundamental departure from traditional exercise prescription approaches used in previous oncology exercise interventions. While established programs such as the Bizi Orain Hybrid Exercise Program [26], LIVESTRONG at the YMCA [46], the PERFECT study protocol [47], and Cormie et al.‘s community-based program [21] primarily utilize percentage-based or repetition maximum-based training models, our protocol incorporates real-time movement velocity as the primary metric to quantify training intensity and regulate fatigue. Campbell et al.‘s exercise guidelines for cancer survivors [13] emphasize the importance of individualized prescription but rely on conventional methods (percentage of 1RM, RPE) for prescription parameters. In contrast, the SPoRT-PCa-ADT program uses mean propulsive velocity (MPV) as a prescription variable that offers an alternative approach to individualizing exercise. MPV may be particularly relevant for ADT patients who experience significant variability in day-to-day performance capacity due to treatment-related fatigue and side effects.

The 24-week duration of the SPoRT-PCa-ADT Program exceeds the typical 12-week interventions seen in studies such as PERFECT [47], Bizi Orain [1], and the BEAUTY program for breast cancer patients [48]. This extended timeframe allowed us to implement a three-phase periodization structure (adaptation, development, and maintenance), which differs substantially from the progression models in previous studies. For example, the Bizi Orain program [26] utilizes a monthly progression model with increasing intensity through changes in HRR percentages and duration of intervals, while our protocol systematically progresses training load, repetition targets, and velocity loss thresholds across distinct phases. Haas et al.‘s FitSTEPS for Life program [49] offers ongoing exercise support without a formalized periodization structure, whereas the SPoRT-PCa-ADT Program’s clearly defined phases with specific load, volume, and intensity parameters represent a more structured approach to progression. This difference reflects varying philosophical approaches to exercise programming, emphasizing systematic overload principles common in sports science that have been less frequently applied in cancer exercise interventions.

The exercise protocol of SPoRT-PCa-ADT Program primary focus on the squat exercise differs markedly from the more diverse exercise selection employed in most previous studies. Galvão et al. [20] and Newton et al. [50] utilized comprehensive resistance training programs targeting all major muscle groups with 8–10 exercises per session. Similarly, the CanWell program described by Cheifetz et al. [51] incorporated a variety of resistance exercises addressing multiple muscle groups. Our more focused approach was selected based on the specific pattern of muscle loss observed in ADT patients (particularly affecting lower extremity function) and the high transfer of squat strength to activities of daily living. This selective approach contrasts with the more generalized whole-body resistance training commonly used in cancer exercise programs but aligns with research suggesting that prioritizing large muscle group exercises may be particularly effective for countering muscle loss [52, 53].

A distinctive feature of the SPoRT-PCa-ADT Program compared to previous interventions is the precision of training intensity prescription and monitoring. While most previous programs use relatively broad intensity ranges (e.g., RPE 12–16 in Bizi Orain [26], 60–85% 1RM in Galvão et al. [20]), our protocol specifies precise velocity targets (0.92 m/s corresponding to 60% 1RM) and velocity loss thresholds (10%) that provide objective measures of both absolute intensity and fatigue accumulation within sessions. This approach differs fundamentally from the intensity prescription methods in studies like Winters-Stone et al. [52], which prescribed resistance training at 60–70% 1RM based on periodic 1RM testing, and Taaffe et al. [53], which utilized 6-12RM loads progressed when participants could perform more than the prescribed repetitions. The real-time velocity feedback in the exercise training allows for daily autoregulation of load based on performance capacity, rather than relying on predetermined progressions or subjective assessments of effort. The daily readiness assessment incorporated in the SPoRT-PCa-ADT Program (including resting heart rate, subjective fatigue ratings, and submaximal movement velocity) represents another methodological difference from previous studies. While most exercise oncology interventions rely on general guidelines for progression and modification, few have implemented systematic readiness assessments to guide day-to-day adjustments in training prescription, as recommended on exercise prescription for clinical populations [15].

The implementation model of the SPoRT-PCa-ADT Program (collaboration between a specialized sports center and a hospital-based urology department) represents a hybrid approach between clinical and community settings. This differs from both hospital-based programs like the BEAUTY program [48] and purely community-based approaches like LIVESTRONG at the YMCA [46], or FitSTEPS for Life [54]. Our approach most closely resembles the model described by Cormie et al. [21, 55] which utilized exercise physiologists in community settings with medical oversight. However, while Cormie et al.‘s program employed general exercise physiologists, the SPoRT-PCa-ADT Program specifically utilized sports scientists with specialized training in velocity-based methodologies, reflecting the technical demands of the intervention approach. The supervised nature of our program (twice weekly with monitoring of specific performance parameters) contrasts with more self-directed approaches such as the home-based components described in the PERFECT study [47] and Hojan et al. [56]. While supervised exercise is generally associated with better outcomes in cancer patients [57, 58], the resource requirements of such approaches must be considered in the context of scalability and implementation.

The assessment methods used in the SPoRT-PCa-ADT Program include several measures common to exercise oncology research (400-meter walk test, chair rise test, anthropometry) but also incorporate specialized assessments not typically included in previous studies. The use of movement velocity measurements (mean propulsive velocity) and detailed force-time analysis during isometric testing provides more comprehensive assessment of neuromuscular function than the strength and functional assessments typically employed in previous research. For example, while the PERFECT study [47] utilizes VO2peak and muscle strength as primary physical fitness outcomes, our protocol’s inclusion of velocity-based metrics and rate of force development measures offers potential insights into neuromuscular adaptations that may be particularly relevant to functional performance in daily activities. This expanded assessment approach aligns with recommendations from Stout et al. [18] regarding comprehensive evaluation of exercise effects in cancer patients.

While our protocol focuses specifically on locally advanced or metastatic prostate cancer, with or without previous local treatment, and metastatic stages, other programs have addressed different cancer populations or treatment phases. For instance, the Bizi Orain program [26] includes patients with any cancer diagnosis, while the PERFECT study [47] focuses on esophageal cancer patients. The BEAUTY program [48] specifically targets breast cancer patients undergoing treatment, and LIVESTRONG at the YMCA [54] serves diverse cancer types. The specific physiological challenges of ADT informed several aspects of our protocol design that differ from programs targeting other populations. The emphasis on preserving muscle mass and strength through substantial lower body resistance training addresses the accelerated muscle loss associated with ADT, which may not be as pronounced in other cancer populations.

The SPoRT-PCa-ADT Program’s close collaboration between exercise professionals and the urology department represents a more integrated approach than some previous studies. While many exercise oncology programs operate relatively independently from oncology care, our protocol incorporated regular communication between exercise physiologists and medical providers, with standardized reporting pathways for adverse events or concerning symptoms. This integrated approach parallels recommendations from Schmitz et al. [22] regarding exercise as medicine in oncology care but differs from the more separate parallel care models described in some community-based programs. The formalized evaluation methodology utilized in our protocol, with results reported back to referring oncologists, further distinguishes our approach as an integrated component of supportive care rather than an adjunctive service.

Unlike many previous exercise oncology interventions that have primarily employed single-group pre-post designs, the inclusion of a concurrently assessed control group in the present study enhances the methodological rigor by allowing for a direct comparison of the specific efficacy of the supervised SPoRT-PCa-ADT program against a home-based exercise approach. This design strengthens the evidence base by controlling for time-related effects and general support interventions.

## Supplementary Information

Supplementary Material 1.

## Data Availability

No datasets were generated or analysed during the current study.

## Abbreviations

ADT: Androgen Deprivation Therapy
BFI: SF-Brief Fatigue Inventory Short form
BMI: Body Mass Index
CMJ: Countermovement Jump
FACT: G-Functional Assessment of Cancer Therapy-General
FACT: P-Functional Assessment of Cancer Therapy-Prostate
HADS: Hospital Anxiety and Depression Scale
Hb: Hemoglobin
HRR: Heart Rate Reserve
hsCRP: high-sensitivity C-reactive protein
IL: 6-Interleukin-6
IPSS: International Prostate Symptom Score
LDH: Lactate Dehydrogenase (note: manuscript lists as alkaline phosphatase levels)
LNR: Lymphocyte-to-Neutrophil Ratio
MICE: Multiple Imputation by Chained Equations
MIF: Maximum Isometric Force
MPV: Mean Propulsive Velocity
MVIC: Maximum Voluntary Isometric Contraction
PCa: Prostate Cancer
PCS: Prostate Cancer Subscale
PSQI: Pittsburgh Sleep Quality Index
PSA: Prostate-Specific Antigen
RCT: Randomized Controlled Trial
RFD: Rate of Force Development
RFDmax: Maximum Rate of Force Development
RM: Repetition Maximum (as in 1RM = 1-Repetition Maximum)
RPE: Rating of Perceived Exertion
SOPs: Standard Operating Procedures
SPoRT-PCa-ADT: Standardized Program of Resistance Training for Prostate Cancer Patients Receiving Androgen Deprivation Therapy
SQ: Squat
VBT: Velocity-Based Training
VO2peak: Peak Oxygen Consumption
